# Postoperative extended-volume external-beam radiation therapy in high-risk esophageal cancer patients: a prospective experience

**DOI:** 10.3747/co.v16i4.355

**Published:** 2009-08

**Authors:** E. Yu, P. Tai, J. Younus, R. Malthaner, P. Truong, L. Stitt, G. Rodrigues, R. Ash, R. Dar, B. Yaremko, A. Tomiak, B. Dingle, M. Sanatani, M. Vincent, W. Kocha, D. Fortin, R. Inculet

**Affiliations:** * Departments of Radiation Oncology (Ash, Dar, Rodrigues, Yaremko, Yu), Medical Oncology (Dingle, Kocha, Sanatani, Vincent, Younus), Biostatistics (Stitt), Surgical Oncology (Fortin, Inculet, Malthaner), London Regional Cancer Program, London Health Sciences Centre, University of Western Ontario, London, ON; † Department of Radiation Oncology, Allan Blair Cancer Center, Regina, SK; ‡ Department of Radiation Oncology, BC Cancer Agency, Vancouver Island Center, University of British Columbia, Victoria, BC; § Department of Medical Oncology, Kingston Regional Cancer Center, Kingston, ON

**Keywords:** Pilot study, cancer, esophagus, extended volume, irradiation

## Abstract

**Background and Purpose:**

Extended-volume external-beam radiation therapy (rt) following esophagectomy is controversial. The present prospective study evaluates the feasibility of extended-volume rt treatment in high-risk esophagectomy patients with a cervical anastomosis receiving postoperative combined chemoradiation therapy.

**Patients and Methods:**

From 2001 to 2006, 15 patients with resected esophageal cancer were prospectively accrued to this pilot study to evaluate the adverse effects of extended-volume rt. Postoperative management was carried out at London Regional Cancer Program. Eligibility criteria were pathology-proven esophageal malignancy (T3–4, N0–1), disease amenable to surgical resection, and esophagectomy with or without resection margin involvement. Patients with distant metastases (M1) and patients treated with previous rt were excluded. All 15 study patients received 4 cycles of 5-fluorouracil–based chemotherapy. External-beam rt was conducted using conformal computed tomography planning, with multi-field arrangement tailored to the pathology findings, with coverage of a clinical target volume encompassing the primary tumour bed and the anastomotic site in the neck. The radiation therapy dose was 50.40 Gy at 1.8 Gy per fraction. The rt was delivered concurrently with the third cycle of chemotherapy. The study outcomes—disease-free survival (dfs) and overall survival (os)—were calculated by the Kaplan–Meier method. Treatment-related toxicities were assessed using the U.S. National Cancer Institute’s Common Toxicity Criteria.

**Results:**

The study accrued 10 men and 5 women of median age 64 years (range: 48–80 years) and TNM stages T3N0 (*n* = 1), T2N1 (*n* = 2), T3N1 (*n* = 11), and T4N1 (*n* = 1). Histopathology included 5 adenocarcinomas and 10 squamous-cell carcinomas. Resection margins were clear in 10 patients. The median follow-up time was 19 months (range: 3.5–53.4 months). Before radiation therapy commenced, delay in chemotherapy occurred in 20% of patients, and dose reduction was required in 13.3%. During the concurrent chemoradiation therapy phase, 20% of the patients experienced chemotherapy delay, and 6.6% experienced dose reduction. No patient experienced treatment-related acute and chronic esophagitis above grade 2. Disease recurred in 40% of the patients (6/15), and median time to relapse was 24 months. No tumour recurred at the anastomotic site. The median dfs was 23 months, and the median os was 21 months.

**Conclusions:**

Extended-volume external-beam rt encompassing the tumour bed and the anastomotic site is feasible and safe for high-risk T3–4, N0–1 esophageal cancer patients after esophagectomy.

## 1. INTRODUCTION

Surgery has traditionally been considered the standard therapy for localized esophageal cancer 1. Post-operative radiation therapy (rt) 2,3 and postoperative chemoradiation 4–6 have been used for esophageal cancer patients deemed to be at high risk for recurrence after esophagectomy. As compared with surgery alone, postoperative chemotherapy and rt 7,8 have been reported to be beneficial with regard to survival.

Optimal rt target-volume definition in esophageal cancer patients receiving primary chemoradiation is controversial 9, partly because of the significant changes in patient anatomy and function, and partly because of the limited information available to evaluate the benefit on anastomotic site coverage in the postsurgical rt target volume. The literature contains limited retrospective or prospective information regarding the necessity for irradiation of the anastomosis and regarding the safety of the relatively large treatment volume when combined with chemotherapy postoperatively. We previously reported a retrospective analysis of extended-volume external-beam rt with a clinical target volume (ctv) covering the anastomotic site in post-esophagectomy high-risk patients 10. The present study was our local prospective pilot trial assessing the feasibility of incorporating the extended-volume regimen into rt planning for a postsurgical adjuvant trial in esophageal cancer.

## 2. PATIENTS AND METHODS

From 2001 to 2006, we prospectively accrued 15 patients with resected esophageal cancer to this pilot study at the London Regional Cancer Program to evaluate the adverse effects of postoperative extended-volume rt delivered with concurrent chemotherapy and of the complications of extended-volume rt concurrent with chemotherapy. Eligibility criteria were pathology-proven esophageal malignancy; 2002 American Joint Committee on Cancer 11 stage T3–4, N0–1 disease; and esophagectomy with or without resection margin involvement. Patients with distant metastasis (M1), those who had received previous rt, or those who declined to sign a consent form were excluded.

Adjuvant therapy consisted of chemotherapy followed by concurrent chemoradiation 4 weeks post surgery or as soon as the patient recovered from the surgical procedure. Chemotherapy consisted of 4 cycles of epirubicin 50 mg/m^2^ day 1, 5-fluorouracil 200 mg/m^2^ continuous infusion for 21 days, and cisplatin 60 mg/m^2^ day 1, repeated every 21 days, with epirubicin omitted during the concurrent phase with rt.

**FIGURE 1:**
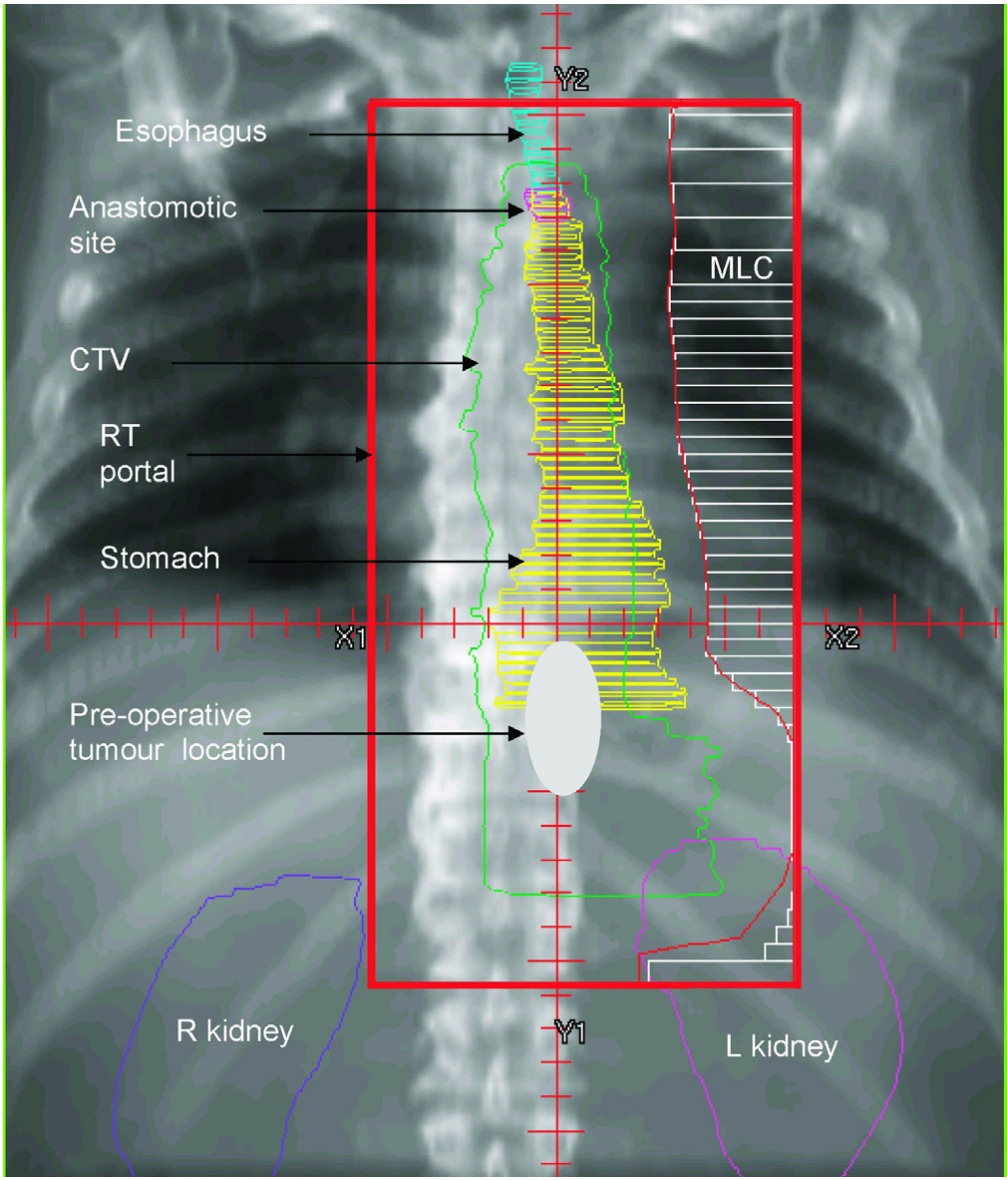
Sample computed tomography simulated radiotherapy (rt) portal for extended-volume rt with coverage of anastomotic site for esophageal cancer after surgery. mlc = multileaf collimator; ctv = clinical target volume; r = right; l = left.

Delivery of the external-beam rt used conformal computed tomography (ct) planning, with a multi-field arrangement tailored to the pathology findings and a ctv encompassing the primary tumour bed and the anastomotic site in the neck. The initial rt target volume was defined with margins of 5 cm above and below the presurgical gross tumour volume, and a 2-cm margin to cover the mediastinal lymph nodes medially and laterally, with the superior margin extended at least 2 cm above the anastomotic site 10 (Figure 1). The boost fields had minimum 2-cm margins around the presurgical target volume. The total rt dose was 50.4 Gy, with 1.8 Gy per fraction (initially 30.6 Gy in 17 fractions, followed by 19.8 Gy in 11 fractions), delivered concurrently with the third cycle of chemotherapy.

Follow-up evaluations of the patients were performed every 3 months during years 1–2, every 4 months during years 3–4, every 6 months during year 5, and annually thereafter. Follow-up investigations included screening blood cell count and chemistries, chest radiography, and ct of chest and abdomen. At the time of relapse, investigations were carried out as clinically indicated and included endoscopy; barium-swallow esophagram; brain, chest, and abdominal ct; and bone scan.

Local relapse was defined as tumour recurrence at or immediately adjacent to the anastomotic site. Regional relapse was defined as recurrence at the mediastinum or peri-esophageal region (or both), excluding local relapse. Distant relapse was tumour recurrence at distant site—for example, brain, liver, or lung.

Treatment–related toxicities were assessed using the U.S. National Cancer Institute (nci ) Common Toxicity Criteria. Acute (90 or fewer days from rt start) side effects of radiation therapy were documented using the revised nci Common Toxicity Criteria 12.

Grade 2 esophagitis related to rt was described as dysphagia, requiring predominantly pureed, soft, or liquid diet for acute effect; inability to take solid food normally; swallowing semisolid food; and need for dilation for late effect. Late rt toxicity is defined as an adverse event occurring more than 90 days from rt start, according to the Late Radiation Morbidity Scoring Scheme from the Radiation Therapy Oncology Group (rtog) and the European Organization for Research and Treatment of Cancer, and the nci 1999 Common Toxicity Criteria. Late gastrointestinal rt morbidity (rtog grading system)—grade 2 toxicity described as moderate diarrhea and colic with bowel movement more than 5 times daily, or intermittent bleeding—was also analyzed.

For the purpose of the study, the marker for any toxicity-related treatment break was measured by the length (in days) of the interruption in the chemotherapy or rt schedule arising during the concurrent phase of chemoradiation treatment. Hematologic criteria for interruptions during concurrent chemotherapy rt included absolute neutrophil count below 1000/mm^3^; neutropenic fever or sepsis; thrombocytes below 80,000/mm^3^. Locoregional symptomatology for treatment interruption included severe esophagitis (that is, severe odynophagia or dysphagia, intolerable pain), impaired nutrition with nausea and vomiting; and dehydration requiring hospitalization 10.

Overall survival (os) was defined as the interval between the date of pathology diagnosis and death or last follow-up, with any death being defined as an event. Disease-free survival (dfs) was defined as the interval between the date of pathology diagnosis and the date of first recurrence or last follow-up, with recurrence treated as an event. Survival estimates were obtained using the Kaplan–Meier method 13.

## 3. RESULTS

Table I summarizes the characteristics of the study cohort. The 15 subjects (10 men, 5 women) had a median age of 64 years (range: 48–80 years). The TNM stages in the group were T3N0 (*n* = 1), T2N1 (*n* = 2), T3N1 (*n* = 11), and T4N1 (*n* = 1). Histopathology included 5 adenocarcinomas and 10 squamous-cell carcinomas. Surgery was either transhiatal (87%) or transthoracic (13%), with clear resection margins in 10 patients and a close or positive radial resection margin in 5 (Table I). The median follow-up was 19 months (range: 3.5–53.4 months).

Table II summarizes the treatment characteristics in the patient cohort. Before the start of rt, delay in chemotherapy and chemotherapy dose reduction occurred in 20% and 13.3% of the patients respectively. During concurrent chemoradiation, these proportions were 20% and 6.6%.

These reasons were cited for chemotherapy delay:

- Cycle 1: physician and patient choice
- Cycle 2: febrile neutropenia, diarrhea
- Cycle 3 (with rt): neutropenia
- Cycle 4 (with rt): neutropenia and patient choice

These reasons were cited for chemotherapy dose reduction:

- Cycle 1: no dose reductions
- Cycle 2: febrile neutropenia, diarrhea, and physician choice
- Cycle 3 (with rt): febrile neutropenia, mucositis, hand–foot syndrome
- Cycle 4 (with rt): patient choice

No rt delays or rt dose reductions occurred in this patient cohort.

During the concurrent chemoradiation treatment period, 1 patient experienced grade 1 esophagitis, and 1 patient, grade 2 nausea and vomiting. The constitutional symptoms observed were moderate taste alteration in 2 patients and poor appetite in 3 patients.

During the follow-up period 90 or fewer days from rt start, 2 patients had grade 1 cough, 1 patient had grade 1 nausea and vomiting, 1 patient had grade 1 esophagitis, 1 patient had grade 2 nausea and vomiting, and 1 patient had grade 2 diarrhea and abdominal cramps. The constitutional symptoms observed were mild-to-moderate taste alteration in 2 patients and mild poor appetite and low energy level in 3 patients.

During the follow-up period more than 90 days from rt start, 2 patients had grade 1 shortness of breath; 1 patient had grade 2 pneumonitis; 2 patients had grade 2 dysphagia, difficulty with solid food requiring esophagoscopy and dilatation for anastomotic strictures; and 2 patients had grade 1 nausea and vomiting. The constitutional symptoms observed were grade 1 fatigue (increase in fatigue over baseline, but not altering normal activities) in 2 patients, moderate taste alteration in 1 patient, and mild poor appetite in 1 patient (Table III).

No treatment-related esophagitis or pneumonitis of greater than grade 2 was observed during treatment and in the follow-up assessments. No chemoradiation treatment–related mortality occurred in the study cohort: specifically, no anastomotic leaks or wound breakdown occurred.

**FIGURE 2:**
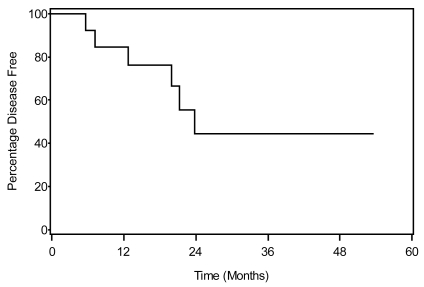
Disease-free survival in the study population to date.

Disease recurrence was observed in 40% (6/15) of the patients, with median time to relapse being 24 months (Figure 2). No tumour recurrence at the anastomotic site was observed. Relapses were exclusively distant metastases, with lung and liver as the most common sites (Table IV). The median, 1-year, and 2-year dfs and os rates were 23 months, 80%, and 44% and 21 months, 65%, and 38% respectively.

## 4. DISCUSSION

The results of this pilot study are consistent with a previous retrospective analysis from our institution reporting complications of extended-volume rt 10. The current prospective trial confirmed the absence of grades 3 and 4 adverse effects with the use of extended volume rt concurrent with chemotherapy. Qiao and co-workers 14 reviewed 102 cases of squamous cell carcinoma of the esophagus receiving postoperative rt of 50 Gy, in which 43 patients treated with extended rt fields covering supraclavicular nodes, anastomotic sites, and loco–tumour bed were compared with 59 patients receiving rt to the loco–tumour bed only. Although os differences between the two groups were nonsignificant, treatment side effects and complications were also nonsignificantly different (18.6% vs. 11.9%, *p* = 0.343), including no difference in the degree of severity of nausea and anorexia between the two groups. No anastomotic leakage, fistulae, or local disease recurrence were observed during follow-up.

Benign anastomotic stricture can occur as early as 27 days and up to 11 months (median: 2–3 months) post transhiatal esophagectomy in esophageal cancer. Depending on the surgical technique, the anastomotic stricture rate from some surgical series can be as high as 42% 15–17. Singh and co-workers 17 reported that patients who underwent total mechanical anastomosis experienced anastomotic stricture requiring dilation at a rate of 18%. Our current prospective study found a rate of 13% (2 patients) of anastomotic stricture post esophagectomy while receiving adjuvant chemoradiation.

Although the median follow-up in our patient cohort is 19 months at the time of writing, any additional adverse effects from the development of anastomotic stricture while receiving adjuvant chemoradiation have yet to occur. Distinct from the finding in our previous retrospective study of 15% locoregional relapse with a median follow-up of 30 months after extended-volume rt, the current pilot study, with a shorter median follow-up (19 months), has yet to identify any locoregional recurrence. In the current study, the recurrences (40%) were all in distant sites, with liver and lung being the most common sites. That result is consistent with our previous report that 85% of relapses in patients treated with extended-volume rt are distant failures.

Although some surgical series have reported that anastomotic recurrence rates are as low as under 5% 18,19, autopsy studies of patients with advanced tumours have reported tumour at the anastomosis in almost 30% of cases 20. Consistent with those studies, our previous series reported a 29% anastomotic recurrence rate for high-risk patients in whom the rt volume did not include the anastomotic site.

Xiao *et al.* 21 reported a series of 549 cases of squamous cell carcinoma of the esophagus. Postoperative rt with 50–60 Gy significantly reduced the incidence of intrathoracic recurrence in high-risk patients with positive nodes and also in patients with negative nodes. Our current data are consistent with our previous series and similar to data reported by Xiao *et al.* 21, finding that high-risk patients treated with rt encompassing the anastomosis have not experienced anastomotic site recurrence and that postoperative rt reduces the risk of intrathoracic recurrence.

The optimal dose for postoperative rt in esophageal cancer is also controversial. Much of the information on rt dose has been derived from reports of preoperative management, with a prescribed rt dose of 30 Gy to the regional nodes and a boost of 20 Gy to the primary tumour, delivered with chemotherapy. This regimen is superior to 64 Gy of rt alone without chemotherapy 22. Regimens using a higher rt dose level of 64.8 Gy have been reported not to be superior to 50.4 Gy concurrently with chemotherapy 23. In a single-institution study, Yu *et al.* 10 reported that a postoperative rt dose of 30.6 Gy to anastomotic site and 50.4 Gy to the tumour bed concurrent with chemotherapy is sufficient and significantly reduces recurrence at both the anastomotic site and the tumour bed. Besides esophageal cancer, the management of anal canal cancer 24 has been successful using a rt dose of 30–36 Gy concurrent with chemotherapy for microscopic disease eradication. That report concurs with Byfield 25 that chemotherapy can enhance the local effects of radiation to reduce the likelihood of spread from the primary, but also to reduce or limit micrometastasis. The rt dose level sufficient to reduce relapse when delivered to the tumour bed concurrently with chemotherapy, while still balancing acceptable toxicity, is not yet clear. Total rt doses of 45–50 Gy to the tumour bed have been reported 7,8,26 to be acceptable and are associated with tolerable treatment side effects.

We acknowledge several limitations of the present study, including a small sample size and short follow-up. We also acknowledge the relative rarity of the patient group receiving postoperative chemoradiation for high risk of recurrence when preoperative chemoradiation is recommended as the treatment of choice in many cancer treatment centers 27. However, the findings on treatment toxicity with the use of extended-volume rt encompassing the anastomotic site postoperatively in high-risk cancer patients in the present prospective study confirm our centre’s previously reported retrospective experience that no addition treatment complications—including treatment interruptions and gastrointestinal toxicity— occur with the use of extended-volume rt 10.

The management of esophageal carcinoma, particularly with esophageal rt, has advanced since the end of the 1990s. A single treatment modality for patients with locoregional disease has now been demonstrated to be suboptimal 28,29. Tri-modality treatment offers the potential to improve outcomes for patients with high-risk disease. In employing tri-modality therapy, the controversy with respective to whether surgery should be performed before or after chemoradiotherapy remains a topic of debate. The current study demonstrating the safety and feasibility of extended-volume rt and the associated encouraging early results in locoregional control suggest that this rt regimen may be incorporated into future studies comparing neoadjuvant chemoradiotherapy with adjuvant chemoradiotherapy trimodality management.

## 5. CONCLUSIONS

Extended-volume external-beam rt encompassing the tumour bed and anastomotic site is feasible and safe for high-risk T3–4, N0–1 esophageal cancer patients post esophagectomy.

## Figures and Tables

**TABLE I:** Patient demographics

Age (years)	
Median	64
Range	48–80
Sex	
Male	10
Female	5
Stage	
T3N0	1
T2N1	2
T3N1	11
T4N1	1
Tumour pathology	
Squamous-cell	10
Adenocarcinoma	5
Margin status	
Clear	10
Close/positive	5

**TABLE II:** Chemotherapy delay and dose reduction (dr) in the study patients

*Patient id*	*Chemotherapy cycle*							
	*1*		*2*		*3*+*rt*		*4*+*rt*	
	*Delay*	*dr*	*Delay*	*dr*	*Delay*	*dr*	*Delay*	*dr*
1	−	−	−	−	−	−	−	−
2	−	−	−	−	+	−	+	+
3	−	−	−	−	−	−	−	−
4	−	−	−	−	+	−	+	−
5	−	−	−	−	−	+	−	−
6	+	−	−	+	−	−	−	−
7	−	−	+	−	−	+	−	−
8	−	−	−	−	−	−	−	−
9	−	−	+	+	−	−	+	−
10	−	−	+	−	+	−	−	−
11	−	−	−	−	−	+	−	−
12	−	−	−	−	−	−	−	−
13	−	−	−	−	−	−	−	−
14	−	−	−	−	−	−	−	−
15	−	−	−	−	−	−	−	−
Overall (%)	6.6	0	20	13.3	20	20	20	6.6

id = identification number; rt= radiotherapy.

**TABLE III:** Side effects and complications in the study patients

*Adverse events*	*Days from radiotherapy start*							
	≤*90* *Event grade*^a^				>*90* *Event grade*^b^			
	*1*	*2*	*3*	*4*	*1*	*2*	*3*	*4*
Lung^c^ [*n* (%)]	2 (13)	0	0	0	2 (13)	1 (7)	0	0
Small/large bowel^d^ [*n* (%)]	1 (7)	2 (13)	0	0	2 (13)	0	0	0
Esophagus^e^ [*n* (%)]	1 (7)	0	0	0	0	2 (13)	0	0
Constitutional symptoms^f^ [*n* (%)]	3 (20)	2 (13)	0	0	3 (20)	1 (7)	0	0

a According to the U.S. National Cancer Institute Common Toxicity Criteria.

b According to Radiation Therapy Oncology Group/European Organization for the Research and Treatment of Cancer late radiation morbidity scoring.

c Pneumonitis, cough.

d Nausea, diarrhea.

e Esophagitis, dysphagia.

f Taste alteration, poor appetite, fatigue.

**TABLE IV:** Pattern of relapse and sites of distal relapse in the study patients

*Pattern of first relapse (*N *= 15)*	
Site [*n* (%)]	
Anastomotic site only	0 (0)
Regional only	0 (0)
Distant only	6 (40)
*Distal sites of first relapse (*N *= 6)*	
Organ [*n* (%)]	
Liver	3 (50)
Lung	3 (50)
Brain	2 (33)
Pleura	1 (17)
Kidney (adrenals)	1 (17)
Abdomen	1 (17)
Bone	1 (17)
